# Advances in Targeting the Innate and Adaptive Immune Systems to Cure Chronic Hepatitis B Virus Infection

**DOI:** 10.3389/fimmu.2019.03127

**Published:** 2020-02-07

**Authors:** Zhongji Meng, Yuanyuan Chen, Mengji Lu

**Affiliations:** ^1^Institute of Biomedical Research, Taihe Hospital, Hubei University of Medicine, Shiyan, China; ^2^Institute of Virology, University Hospital Essen, Essen, Germany

**Keywords:** immunotherapy, hepatitis B virus, innate immunity, therapeutic vaccination, T cell therapy

## Abstract

“Functional cure” is being pursued as the ultimate endpoint of antiviral treatment in chronic hepatitis B (CHB), which is characterized by loss of HBsAg whether or not anti-HBs antibodies are present. “Functional cure” can be achieved in <10% of CHB patients with currently available therapeutic agents. The dysfunction of specific immune responses to hepatitis B virus (HBV) is considered the major cause of persistent HBV infection. Thus, modulating the host immune system to strengthen specific cellular immune reactions might help eliminate HBV. Strategies are needed to restore/enhance innate immunity and induce HBV-specific adaptive immune responses in a coordinated way. Immune and resident cells express pattern recognition receptors like TLRs and RIG I/MDA5, which play important roles in the induction of innate immunity through sensing of pathogen-associated molecular patterns (PAMPs) and bridging to adaptive immunity for pathogen-specific immune control. TLR/RIG I agonists activate innate immune responses and suppress HBV replication *in vitro* and *in vivo*, and are being investigated in clinical trials. On the other hand, HBV-specific immune responses could be induced by therapeutic vaccines, including protein (HBsAg/preS and HBcAg), DNA, and viral vector-based vaccines. More than 50 clinical trials have been performed to assess therapeutic vaccines in CHB treatment, some of which display potential effects. Most recently, using genetic editing technology to generate CAR-T or TCR-T, HBV-specific T cells have been produced to efficiently clear HBV. This review summarizes the progress in basic and clinical research investigating immunomodulatory strategies for curing chronic HBV infection, and critically discusses the rather disappointing results of current clinical trials and future strategies.

## Introduction

The rate of newly acquired hepatitis B virus (HBV) infection is well-controlled by prophylaxis with conventional HBsAg vaccines; however, the vast reservoir of nearly 300 million chronic HBV-infected individuals worldwide still represents a serious threat to humans, leading to up to about 900,000 deaths every year (1–3). Persistent HBV infection could result in liver cirrhosis and/or failure, and liver cancer, accounting for most end-stage liver diseases (4, 5).

Currently, PEGylated interferon-α (PEG-IFN-a) and nucleos(t)ide analogs (NUCs) are available antiviral drugs for the effective treatment of chronic HBV infection (5–8). NUCs, with daily oral administration, are widely welcomed by chronic hepatitis B (CHB) patients, and suitable for individuals with liver cirrhosis, liver failure, and pregnancy, due to their excellent safety profiles. Although NUCs can control HBV replication profoundly, and reduce the HBV associated end-stage liver disease and liver cancer, hepatitis B surface antigen (HBsAg) clearance, suppression, and/or seroconversion seldom occur in patients administered monotherapy with NUCs. The discontinuation of NUC treatment might result in liver flare or failure. Thus, an uncertain or even lifelong period of NUC treatment may be needed for most patients with chronic HBV infection. Alternatively, PEG-IFN-α treatment can lead to HB e-antigen (HBeAg) clearance and HBsAg seroconversion in 10–30% of cases within a definite duration of therapy (6, 9, 10). Beside direct antiviral effects, the immunomodulatory property of IFN-α may ultimately induce an immune control of HBV. Meanwhile, poor tolerability with frequent severe undesirable effects and the requirement for subcutaneous administration limit PEG-IFN-a application.

Recently, “functional cure,” which is characterized by loss of HBsAg whether or not anti-HBs antibodies are detected, is becoming an accessible ideal endpoint of antiviral treatment in CHB (3, 11). “Functional cure” represents continued suppression of the activity of covalently closed circular DNA (cccDNA) in the patient liver without the serum markers of viral replication. The remaining cccDNA may be reactivated once the immune system is deeply damaged, leading to the recurrence of hepatitis B. Thus, cccDNA eradication is being pursued intensively as an ultimate therapeutic goal. It is believed that host immune control of HBV infection implies complete elimination or functional inactivation of HBV cccDNA though the underlying molecular mechanisms are not fully understood (12). Based on this assumption, enhancing host immunity to HBV is rationally an attractive approach to cure chronic HBV-infected patients.

In this review, we summarized the available information about strategies for enhancing host innate and adaptive immunity for controlling HBV infection. The relevant basic research resulting from preclinical studies is reviewed in sections enhancing innate immunity to establish an antiviral state: results from preclinical studies and induction of HBV-specific immune responses. The available results of clinical trials are presented in sections current clinical trials based on immunotherapy and IFN-α-based immunotherapy plays an important role in HBV “cure” in individuals with functional intrinsic immune responses. A critical consideration of the results of current clinical trials and discussion about the future strategies are included in section conclusions and future perspectives.

## Immune Pathogenesis of Persistent HBV Infection

The molecular mechanisms accounting for HBV persistence are not fully elucidated. It is generally accepted that dysfunctional immune responses play an essential role in persistent HBV infection as well as liver inflammation, when comparing the characteristics of immune responses in acute hepatitis B and CHB (3, 13–16). Immune responses during CHB are characterized by (1) dysfunctions and exhaustion of HBV-specific CD4^+^ and CD8^+^ T cells (2, 17–19) decreased numbers and dysfunction of DCs and NKs/NKTs (3, 13, 20–23) up-regulated/enhanced expression of regulatory factors, including the immune checkpoint proteins PD-1, CTLA-4, and T cell immunoglobulin domain and mucin domain-3 (Tim-3) (24–26); and (4) impaired innate immune response, especially toll-like receptor (TLR) downregulation and dysfunction (27–32).

To maintain homeostasis, the hepatic immune system preferentially induces tolerance to antigens flushed from the portal vein. In CHB, the suppressive mechanisms in the liver regulate and inhibit T cell functions. It has been confirmed that intrahepatic inflammatory reactions induce multiple suppressive pathways *in situ* in the liver, leading to T cell function suppression (25). Enzymes such as arginase (33) and IDO (34) are released by damaged hepatocytes and cause depletion of amino acids, which are important in maintaining T cell functions (35). Arginine depletion leads to reduction of CD3ζ levels in T cells, subsequently causing TCR-pathway dysfunction (36). Intrahepatic inflammation recruits regulatory T cells (37–41), B cells, and myeloid-derived suppressor cells (42–44), and activate stellate cells, leading to IL-10 and TGF-β production (25). The suppressive events in the liver are vital for protection from severe damage primed by inflammation, while further impairing the functionality of HBV-specific T cells.

In general, high HBV DNA, HBsAg, and HBeAg levels contribute to maintain HBV-specific immune tolerance in chronically HBV-infected individuals. Reduction of both circulating and intrahepatic HBV virions and proteins is a prerequisite for (re-)establishing efficient HBV-specific T-cell responses (45–48). The first evidence that HBV clearance can be achieved by adoptive transfer of bone marrow from anti-HBs-positive donors (49) provides a certain way to cure HBV infection through immune modulation. Liver transplantation may also transfer immune cells from vaccinated donors to recipients, and partially control reinfection of the liver (50). An increasing number of studies have been carried out to explore therapeutic strategies including those involving small molecules to boost HBV immunity in patients, aiming to a functional cure for HBV infection (51–53).

## Therapeutic Strategies for CHB

**Figure 1:**
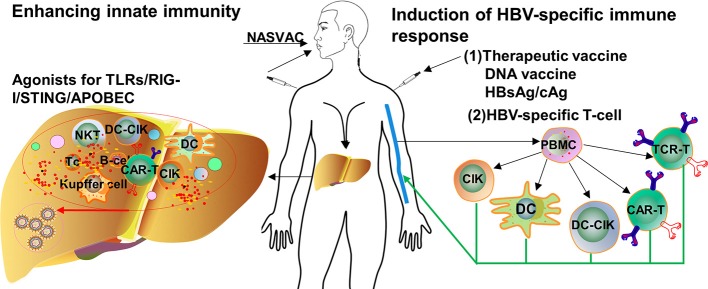
Approaches for the treatment of chronic HBV infection. Available knowledge about HBV immune control and immunopathogenesis; a number of immunomodulatory strategies have been tested to enhance innate and adaptive immunity in preclinical models and clinical trials. TLR, toll-like receptor; RIG-I, retinoic acid-inducible gene 1; STING, stimulator of interferon genes; APOBEC, apolipoprotein B mRNA-editing enzyme catalytic subunit; PBMC, peripheral blood mononuclear cell; DC, dendritic cell; CIK, cytokine-induced killer; CAR-T, chimeric antigen receptor T-cell; TCR, T cell receptor. Dots in various colors indicate different cytokines.

**Figure 2:**
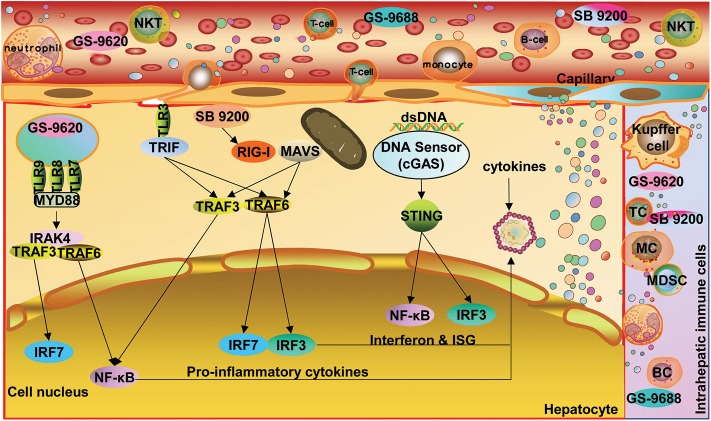
Options for enhancing innate immunity and establish an antiviral state. Oral, intranasal, or subcutaneous application of agonists of PARs, including TLRs, RIG-I, and STING, activates host immune cells and hepatic parenchymal and non-parenchymal cells, leading to the production of IFN and proinflammatory cytokines as well as ISG expression. TLR, toll-like receptor; RIG-I, retinoic acid-inducible gene 1; STING, stimulator of interferon genes; NF-κB, nuclear factor kappa-B; ISG, interferon-stimulated gene; cGAS, cyclic GMP-AMP synthetase. Dots in various colors indicate different cytokines. STING expression in hepatocytes remains controversial.

**Figure 3:**
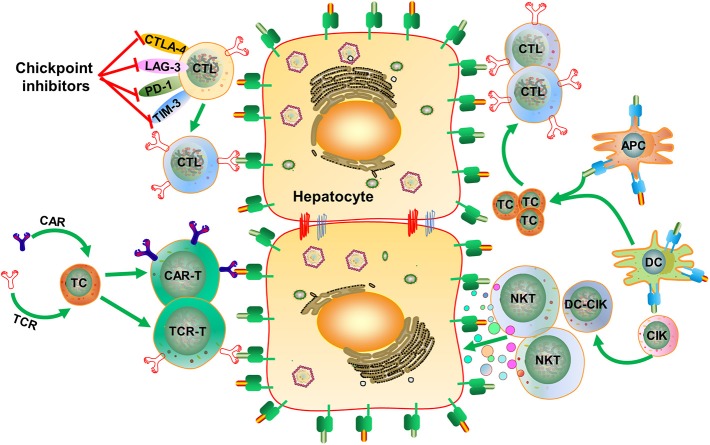
Strategies for inducing HBV-specific immune responses and regaining immunological control of HBV infection. HBV-specific CD8^+^ T cell responses can be induced by therapeutic vaccines, boosted through checkpoint blockade, or renewed by adoptive transfer of *in vitro* activated T/NKT cells or genetically edited HBV-specific CAR-T or TCR-T cells. CTLA-4, cytotoxic T-lymphocyte-associated protein 4; LAG-3, lymphocyte activation gene-3; PD-1, programmed death-1; TIM-3, T-cell immunoglobulin and mucin-domain containing-3; APC, antigen-presenting cell; DC, dendritic cell; CIK, cytokine-induced killer; CAR-T, chimeric antigen receptor T-cell; TCR, T cell receptor. Dots in various colors indicate different cytokines; 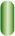, Hepatitis B surface antigen/epitopes; 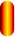 Hepatitis B core antigen/epitopes; 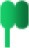, Major Histocompatibility Complex class I molecule; 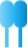, Major Histocompatibility Complex class II molecule.

Based on the knowledge about the immune pathogenesis of chronic HBV infection, a number of innovative strategies may be applied to enhance HBV-specific immune responses in patients (Figure 1). On one hand, oral, intranasal, or subcutaneous application of agonists of pathogen recognition receptors (PRRs), including TLRs, retinoic acid-inducible gene 1 (RIG-I), and stimulator of interferon genes (STING), activates host immune cells and hepatocytes/non-parenchymal liver cells, leading to the production of IFN/expression of interferon-stimulated genes (ISGs) and proinflammatory cytokines, which jointly mount an antiviral state (Figure 2). On the other hand, HBV-specific CTLs can be induced by therapeutic vaccines, boosted through checkpoint blockade, or renewed by adoptive transfer of *in vitro* activated T/NKT cells or genetically edited HBV-specific T cells such as chimeric antigen receptor T (CAR-T) or T cell receptor (TCR)-T cells (Figure 3). These strategies have been explored in the past years. Though their potential usefulness is partly proven, many obstacles hindering the clinical use of these approaches are still to be overcome in the future.

## Enhancing Innate Immunity to Establish an Antiviral State: Results From Preclinical Studies

### TLR Ligands

TLRs play an important role in the innate immune response through sensing viral and bacterial PAMPs and bridging to adaptive immunity. TLRs are widely expressed in immune cells, hepatocytes, and non-parenchymal liver cells (NPCs), which contribute to immune control of HBV (32, 51, 52, 54, 55).

Isogawa and collaborators firstly demonstrated that single application of ligands specific to TLRs-3, 4, 5, 7, and 9 trigger non-cytopathic, IFN-dependent suppression of hepatic HBV replication in HBV transgenic mouse models within 24 h (56). These interesting results ignited hope in treating chronic HBV infection by activating TLR-dependent signaling pathways. Thereafter, various TLR ligands have been tested in cell and animal models with HBV replication, as reviewed previously (52, 57, 58). Direct application of TLR ligands can potently inhibit HBV replication in primary hepatocytes and hepatoma cells through IFN-dependent and -independent pathways. TLR-2 and−4 activation triggers IFN-independent pathways and leads to a robust inhibition of hepadnaviral replication by various intracellular pathways in hepatoma cells and woodchuck hepatocytes harboring woodchuck hepatitis virus (WHV) (57). On the other hand, stimulating NPCs (KCs and LSECs) and DCs with TLR ligands could induce a panel of antiviral mediators (e.g., type I IFN), which inhibit HBV replication *in vitro* (59, 60). Thus, TLR stimulation may not only activate resident parenchymal cells, NPCs, and infiltrated immune cells in the liver but also recruit circulating immune cells to establish an antiviral state (Figure 2). Recently, TLR stimulation has also been found to directly promote T cell functionality via metabolic regulation, adding to its capacity for immunomodulation (61–65). Meanwhile, systemic inflammatory responses after TLR stimulation should be taken into account.

In woodchucks chronically infected with WHV, weekly subcutaneous injection of CpG ODN for 16 weeks induces IFN synthesis with transient and weak viral inhibition (55). When combined with entecavir, potent inhibition of WHV was evidenced by early viral responses and a significant decrease in serum woodchuck hepatitis surface antigen (WHsAg). However, WHsAg seroconversion was not attained (55). Further data analysis suggested that CpG ODN application enhances viral suppression by antiviral treatment.

GS-9620, a TLR7 ligand, has shown great therapeutic potential in woodchuck and chimpanzee models (54, 57). In chronically WHV-infected woodchucks, GS-9620 monotherapy for 4–8 weeks led to continued, significant decrease of serum WHV DNA and sustained WHsAg loss after cessation of treatment in 13/15 animals. Moreover, 7 of the 13 animals developed an antibody response against WHV surface antigen (66). In chronically HBV-infected chimpanzees, reduced HBV viral load and serum HBsAg level were recorded but without HBsAg loss (67). A recent study showed that GS-9620 could induce multiple HBV suppressive factors in human PBMCs, leading to prolonged type I IFN-associated HBV suppression in primary human hepatocytes (PHH) and HepaRG cells, as well as enhanced biosynthesis of immunoproteasome subunits and display of an immunodominant viral peptide in PHH with HBV-infection. The latter may promote T cell recognition and activation in the host and viral control, though GS-9620 itself could not reduce cccDNA levels in hepatocyte culture systems (68).

### RIG-I Activator

SB9200, an activator of RIG-I and nucleotide-binding oligomerization domain-containing protein 2 (NOD2), can induce prolonged IFN-α/β and ISG activation in blood/liver in WHV-infected woodchucks, leading to a 3.7 log10 decrease of serum WHV DNA and an 1.6 log10 decline in serum WHsAg when orally dosed with 30 mg/kg for 12 weeks (69). Interestingly, SB 9200 treatment sequentially followed by ETV administration induces a much more potent suppression of WHV, with a 6.4 log10 decrease in serum WHV DNA load and a 3.3 log10 decline in WHsAg level, delaying the recurrence of viral replication. Thus, SB 9200-induced host responses potentiate the antiviral efficacy of NUCs (70).

### STING Activator

An alternative host factor, cyclic GMP-AMP synthetase (cGAS), was reported to be involved in HBV recognition. Indeed, cGAS can recognize HBV DNA and activate its adaptor protein—STING, leading to ISG56 expression and resulting in the suppression of viral assembly (71). Activation of the cGAS-STING pathway by dsDNA or cGAMP markedly inhibits HBV replication in cell and mouse models (72). An agonist of mouse STING, 5,6-dimethylxanthenone-4-acetic acid (DMXAA) significantly induces the expression of ISGs and reduces hepatic HBV DNA production in hydrodynamic HBV mouse models. DMXAA induces a type I IFN-dominated cytokine response, in contrast to TLR agonists which predominantly trigger inflammatory cytokine and chemokine responses (73). A recent study reported that human hepatocytes do not express STING (74). Nevertheless, treatment of HBV-infected hepatoma cells in culture with cGAMP or DMXAA leads to a significant inhibition of HBV replication, evidenced by concentration-dependent reductions in intracellular HBV mRNA, core-associated DNA, and secreted HBsAg, yet without apparent alteration in the amount of cccDNA (75). A splice isoform of MITA/STING, referred to as MITA-related protein (MRP), specifically blocks MITA-associated IFN activation while still inducing the NF-κB pathway. MRP overexpression significantly inhibits HBV replication by activating the NF-κB pathway in a hydrodynamic injection mouse model. MITA/STING deficiency (MITA/STING^−/−^) enhances HBV replication in mice. Moreover, HBV-specific humoral and CD8^+^ T cell responses are reduced in MITA/STING-deficient animals, indicating an important role for MITA/STING in anti-HBV immunity in innate and adaptive responses (76). Altogether, STING might be a potential target for CHB immunotherapy.

### APOBEC-Mediated Deamination

APOBEC-3 enzymes are involved in host innate immunity against HBV. It was firstly reported in 2005 that human APOBEC3 enzymes are able to extensively edit HBV DNA strands via cytidine deamination (77). In HBV-harboring HepAD38 and HepG2.2.15 cells, cytidine deaminases found endogenously could edit 10–25% of the HBV rcDNA genome within the viral capsid (78). Meanwhile, Hsp90 enhances APOBEC-3-mediated DNA deamination activity in HBV (79). Recent reports also indicated that APOBEC-3 enzymes may mediate the antiviral activity of type I IFN and lymphotoxin by cytidine deamination, leading to cccDNA degradation (80).

Preclinical studies revealed that triggering of innate immunity leads to the production of antiviral and inflammatory mediators, with viral suppression to various extents. However, activation of innate immunity alone presumably does not control HBV infection, unless the adaptive branch of host immunity subsequently comes into play. Among the tested agonists, only the TLR7 ligand GS-9620 has been tested in clinical trials (see below). Other candidate drugs are not yet ready for clinical testing. It will be useful to characterize such candidates not only for activating innate immunity but also for their ability to bridge to adaptive immunity, given that the specific immune response to HBV is critical for effective HBV control.

## Induction Of HBV-specific Immune Responses

### Protein/Polypeptide Vaccines

#### HBsAg/preS Vaccine

Conventional HBsAg vaccines failed to achieve a significant therapeutic effect in either preclinical animal models or patients with CHB. This failure was attributed to HBsAg-specific immune tolerance. Using IL-12 as an adjuvant, Zeng et al. showed that HBsAg immunization efficiently reverses systemic tolerance toward HBV proteins, with enhanced HBV-specific CD8^+^/CD4^+^ T cell responses and reduced CD4^+^Foxp3^+^ Treg cell frequency in HBV-harboring mice (81). The majority of animals administered IL-12-based vaccine acquired HBsAg seronegativity, and hepatitis B core antigen (HBcAg) became undetectable in hepatocytes. Meanwhile, preS1-polypeptide has been shown in HBV carrier mice to induce robust immune responses. Anti-preS1 antibody could clear HBV virions and even lead to HBsAg/HBsAb seroconversion through sequential administration of preS1 and HBsAg vaccines (82).

#### HBcAg Vaccine

Markedly elevated frequencies of HBcAg-specific CTLs have been found in CHB cases capable of controlling HBV replication in comparison with those who did not (83). Thus, HBcAg-based vaccines are considered a promising candidate for CHB treatment. In a pilot study, a peptide-based vaccine containing HBcAg amino acids 18–27 in combination with a Th epitope initiated a low-level CTL activity in CHB patients but failed to clear HBV (84). A synthetic HBcAg vaccine, originally designed to reduce the risk of liver tumors by Inovio Pharmaceuticals Inc., was also reported to be highly potent. However, only very limited information about this vaccine candidate is available.

#### HBsAg/HBcAg Compound Vaccines

Therapeutic vaccines comprising HBsAg and HBcAg and the CpG adjuvant have been shown to elicit strong HBsAg/HBcAg-specific humoral responses and balanced Th1/Th2 responses to HBsAg as well as Th1-type responses to HBcAg in wild-type C57BL/6 mice and HBV transgenic animals. Enhanced HBsAg/HBcAg-specific cellular immune responses lead to significantly reduced serum HBsAg levels without liver injury in HBV transgenic mice (85). A particulate vaccine composed of HBsAg, HBcAg, and the adjuvant ISCOMATRIXTM could induce multi-specific and multi-functional T cells in HBV-Tg mice, especially HBc-specific CD8^+^ T cells with elevated IFN-γ, TNF-α, and IL-2 production. Anti-HBsAg titers reached >10,000 IU/L in 7/8 animals after 4 vaccinations. However, titers of circulating HBV DNA decreased in vaccinated HBV-Tg mice after two and four vaccinations although statistical significance was not reached. HBcAg-positive hepatocytes were also dramatically decreased without obvious liver damage (86).

#### Anti-HBsAg Antibody

Antibody-mediated immunotherapy has been assessed in several preclinical and clinical studies but failed to achieve long-lasting HBV suppression. Zhang et al. developed a new monoclonal antibody (mAb) against HBsAg (mAb E6F6) with remarkable effects in the treatment of persistent HBV replication in several mouse models (87). Indeed, a single dose of E6F6 markedly reduced HBsAg and HBV DNA amounts by over 3 logs for many weeks in HBV-transgenic animals. E6F6 could not only potently prevent primary HBV infection but also reduce secondary spread of HBV from infected hepatocytes in the human-liver-chimeric mouse model. After E6F6-based immunotherapy, anti-HBV T-cell response was restored in mice with persistent HBV replication established by hydrodynamic injection. Fcγ receptor-dependent phagocytosis is considered to play the most critical role in E6F6-associated viral immune clearance, independently on ADCC and CDC (88).

#### DNA Vaccines

DNA vaccines encoding HBsAg and HBcAg induce both humoral and cellular immunity against both HBV antigens, constituting a promising approach for the control of HBV infection (89–93). Upon intramuscular or intradermal injection, *in situ* expressed HBsAg and HBcAg in transfected cells such as myocytes and APCs are processed and presented to host immune cells, resulting in specific B and T cell activation (94, 95). Encouraging results were obtained in pre-clinical studies in the mouse and woodchuck models assessing diverse technologies to improve the efficacy of DNA vaccines, including (i) integration of immunostimulatory cytokines (96); (ii) combination with NUCs (97, 98); (iii) prime-boost immunization regimens (98, 99); (iv) electroporation delivery of DNA vaccines (93, 100); and (v) combination with checkpoint inhibition (93, 100).

In a recent study, Chuai et al. vaccinated rhesus macaques using a complex procedure. Four animals received three doses of HBV DNA vaccines encoding HBsAg, PreS1, and HBcAg for priming, followed by two boosts with recombinant vaccinia viral vectors encoding HBsAg, PreS1, and HBcAg, with a final boost using fusion protein including HBsAg and PreS1. Anti-PreS1 antibodies were induced quickly upon initial priming with DNA vaccination, followed by anti-HBsAg and anti-HBcAg antibodies. Upon boosting with recombinant vaccinia, both humoral and cellular immune responses to HBsAg, PreS1, and HBcAg were markedly induced, with HBcAg-specific CTL response being the most robust and durable. Further boosting with the fusion protein maintained the immune responses to all three HBV antigens until week 98 after the first vaccination. These results suggested that incorporation of PreS1 and HBcAg may improve the effects of therapeutic vaccines (101).

#### Vaccines Based on Viral Vectors

As mentioned in the previous section, Kosinska et al. tested therapeutic vaccines based on adenoviral vectors in the mouse and woodchuck models and obtained very promising results (98, 99). Moshkani et al. reported a vesicular stomatitis virus (VSV)-based vaccine platform (102). Using a highly attenuated VSV strain expressing MHBs by either intranasal or intramuscular application, they successfully induced MHB-specific CD8^+^ T cell and humoral responses in naive mice capable of preventing HBV replication after challenge by adeno-associated virus harboring HBV (AAV-HBV). In mice with persistent HBV replication, the VSV-MHB system could also induce significant multi-specific T cell responses, leading to decreased serum and hepatic HBV antigen and DNA levels and transient elevation of serum alanine aminotransferase activity. These data provide evidence for the potential utility of vaccine platforms based on viral vectors as alternative therapeutic vaccines against CHB.

The design and effectiveness of HBV vaccines have been improved over the years. Clearly, HBV-specific T cells have been stimulated by these vaccine candidates in different experimental settings with variable levels of HBV suppression. The most effective vaccines are those based on viral vectors, which show superiority over other types in terms of T cell induction. Nevertheless, other types such as DNA vaccines could be applied repeatedly without limitation and used in combination with viral vectors. Currently, preclinical and clinical studies using the available vaccine candidates have been rather unfruitful, leaving the major question as to whether the currently available HBV vaccines are potent enough for immunotherapeutic approaches. The hurdles to be overcome likely lie in the recruitment of activated immune cells into the liver and the maintenance and amplification of primed HBV-specific immunity within the liver. Thus, combinations of antiviral treatment and additional immunomodulatory drugs including TLR ligands and checkpoint inhibitors may be necessary to achieve effective, long-lasting T cell immunity.

### HBV-Specific T-Cell Therapy

Recently, useful technologies to induce or generate antigen-specific T cells were developed. This is a fast-growing research field with great potential for the treatment of chronic viral infections. HBV-specific T cells could be induced by DC vaccines, boosted by checkpoint blockade, or renewed by adoptive transfer of lab-produced HBV-specific T cells like CAR-T and TCR-T cells (103).

#### DC Vaccines

As the most powerful professional antigen-presenting cells, DCs play vital roles in bridging the innate immunity and the adaptive immunity. Meanwhile, myeloid DCs (mDCs) were found to regulate the functional differentiation of HBV-specific CD8^+^ T cells in immune transfer experiments (104). While PD-1 was identified to mediate functional exhaustion of HBV-specific CD8^+^ T cells, T cell functions could be restored by CD40 stimulation of mDCs. This has been validated from bench to bedside in CHB treatment using HBsAg/HBcAg-pulsed DCs. In HBV transgenic mouse models, HBsAg-pulsed DCs could induce HBsAg-specific immune responses. Interestingly, HBcAg-pulsed DCs induce both HBsAg- and HBcAg-specific T cell responses, leading to loss of HBsAg with anti-HBsAg seroconversion (105, 106).

#### CIK/DC-CIK

Cytokine-induced killer (CIK) cells, produced *ex vivo* via treatment of PBMCs or cord blood mononuclear cells with IFN-γ, anti-CD3 antibody, IL-1, and IL-2, are featured as cells with a mixed T- and NK cell-like phenotype (CD3^+^CD56^+^). CIK cells target infected and cancer cells in both MHC-restricted and MHC-unrestricted manners, inducing rapid and unbiased immune reactions. Therefore, CIK cells attract attention as a potential therapeutic tool in malignancies and viral infections (107, 108).

DC-CIK refers to the co-culture of CIK cells with DCs or sequential adoptive transfusion of autologous DCs and CIKs, engaging the crosstalk between DCs and CIKs. DCs, especially antigen pulsed ones, can stimulate NK cells and initiate antigen-specific T- and B-cell responses (109). Increasing evidence shows that combination of DCs can reduce the frequencies of regulatory T cells in CIK cell cultures and increase the rate of CD3^+^CD56^+^ cells (110).

#### Immune Checkpoint Inhibitors

Blockade of immune checkpoints such as PD-1/PD-L1 signaling may relieve the negative regulation of specific T cells or even revive exhausted T cells. Application of antibodies targeting PD-L1 in woodchucks with WHV infection, combined with ETV administration and DNA immunization, successfully enhanced virus-specific T cells, resulting in continued inhibition of viral replication, production of anti-WHsAg antibodies, and complete viral clearance in certain woodchucks (100). A recent *ex vivo* study showed that HBV-specific CD4^+^ T cells isolated from individuals with HBeAg-negative HBV infection could be activated by OX40 stimulation combined with PD-L1 blockade, with remarkably increased IFN-γ and IL-21 production *in vitro*. Functional boost of HBV-specific CD4^+^ T cells via both treatment with OX40 and PD-1 pathway blockade might be useful in curing CHB (111).

Checkpoint inhibitors alone have shown only limited efficacy in chronic HCV patients (112). Virus-specific CD8^+^ T cells could be restored through PD-1 blockade in latent HBV carriers (113). Similarly, blockade of other checkpoint molecules such as Tim-3 and CTLA-4 can also restore virus-specific CD8^+^ T-cell responses in CHB patients (114, 115).

#### Genetically Edited T Cells (CAR/TCR-T)

Spontaneous HBsAg clearance is observed in more than 90% of adults with acute HBV infection accompanied by strong intrinsic or adaptive immune responses (116, 117). Patients with CHB lack effective T cell responses for viral clearance due to various immune tolerance mechanisms. Current T-cell-based therapy uses different types of engineered T cells, which express predefined antiviral characteristics. The basic principle of this process is the use of a new well-functioning T-cell library to replace or enhance the low or depleted energy T-cell library of the host and target the virus-specific immunodominant epitopes (118, 119). Therefore, this strategy may lead to immunological control of HBV infection in patients and CHB cure in long term. However, there are several points that need to be addressed for establishing its effectiveness and safety for CHB cure. First, strong T-cell-mediated killing may lead to severe liver damage and acute liver failure; secondly, it is necessary to demonstrate that engineered T cells indeed have improved functionalities and are able to remain functional under immune tolerizing conditions in the liver.

CAR-T cells can be engineered to identify antigens in an MHC non-dependent manner, providing a wider range of targets compared with natural T-cells. CAR-T cells show highly effective anti-tumor activities in a variety of tumors, including CD19^+^ acute leukemia, and may be developed into a safe and effective tumor treatment strategy (120). Bohne et al. firstly generated CAR-T cells targeting HBsAg. CAR-T cells directed against the “a” determinant of HBsAg and aa37-43 in the preS1 protein were able to recognize HBsAg-positive primary human hepatocytes and HepG2.2.15 cells, and specifically eliminate HBV-infected target cells (118).

A couple of studies have assessed the *in vivo* effects of CAR-T/TCR-T cells directed to HBV in mouse models (121–124). Kah et al. showed that HBV-TCR-T cells could lyse cultured HBV-harboring hepatoma cells, and viral loads were reduced within 12 days of treatment with three injections of HBV-TCR-T cells in HBV-infected human liver chimeric mice (122). Three reports about HBsAg-CAR-T cells are available. Krebs et al. showed that CD8^+^ T cells expressing HBsAg-specific CARs recognize different HBV subtypes and could be expanded in immunocompetent HBV transgenic mouse models, resulting in efficient control of HBV replication with only transient liver damage (121). Kruse et al. tested HBsAg-CAR T cells in HBV-infected human liver chimeric mice. As a result, an average of 4.7-fold reduction of serum HBsAg, 3.0-fold decrease in viral load, and 70% reduction of HBcAg-positive hepatocytes were found 36 days after adoptive transfer of HBsAg-CAR T cells (123). Meanwhile, human plasma albumin levels were unaltered, suggesting non-cytopathic viral clearance. Festag et al. engineered fully human, second-generation CAR T cells targeting HBsAg and tested them in an AAV-HBV mouse model with specific tolerance to human HBsAg-CAR. In this system, long-lasting antiviral effects were demonstrated with 2 log10 decrease of HBsAg and 60% reduction of HBV-DNA for up to 110 days upon adoptive transfer. However, HBsAg-CAR T cells failed to completely clear HBV in the animals (124). Recently, HBV-specific T cells produced by lymphocytes using HBV-T cell receptor mRNA could reduce the viral load of HepG2.2.15 cells by 50% with no overt liver toxicity (125). Whether HBV-specific T cells could indeed suppress HBV efficiently while not directly killing hepatocytes requires further investigation. The approach with HBV-specific CAR/TCR-T cells represents a technology with great therapeutic potential.

Though DC-CIK based therapy has been applied in patients in China, its systematic analysis has not been performed. It is important to follow up patients administered DC-CIK treatment and determine the actual usefulness of this approach. The recent progress in T-cell-based therapies for tumor treatment is encouraging and provides therapeutic guidance for major chronic viral infections with HIV and HBV. Unlike in tumor treatment, the safety issue in antiviral therapy is significantly stricter and any risk of uncontrolled overshooting immune responses in patients is not acceptable. Similarly, the application of immune checkpoint inhibitors faces similar challenges and needs to balance the enhancement of host immune responses and the control of the risk of undesired immunopathology.

## Current Clinical Trials Based on Immunotherapy

Many immunotherapeutic approaches have been tested in diverse animal models; however, only few of these attempts reached the phase of clinical trials (Table 1). It should be pointed out that combination therapy with potent antivirals and therapeutic DNA and viral-vector-based vaccines was partially successfully tested in the woodchuck model, with complete viral control and induced anti-surface antibody. Yet, similar approaches failed in chronically HBV-infected patients.

**Table 1:** Current clinical trials based on immunotherapy.

**Therapeutics**	**Vaccine/agonist**	**Company/Organization**	**Status**	**References**
GS4774	X, large-S	Gilead Sciences	I-IIs	(126–128)
NASVAC	HBsAg+HBcAg	Clinical Research Organization, Dhaka, Bangladesh	III	(129, 130)
INO-1800	Plasmids encoding HBsAg + HBcAg	Inovio	I	No data available
HepTcell	T cell epitopes	Altimmune	I	(131)
DNA vaccine	preS2 +S	Institut National de la Santé Et de la Recherche Médicale, France French National Agency for Research on AIDS and Viral Hepatitis	I/II	(132, 133)
DNA vaccine	JNJ-64300535	Janssen Sciences Ireland UC	I	No data available
DNA vaccine-HB-110	S+preS+Core+Pol.	Genexine, Inc.	I	No data available
DNA vaccine	UN	PowderMed	I	No data available
DNA vaccine	HBs	Genexine, Inc.	NA	No data available
DNA vaccine	INO-1800+INO-9112	Inovio Pharmaceuticals	I	No data available
TG1050	ADV-S+C+Pol.	Transgene	I	No data available
ChAd155-hIi-HBV	UN	GlaxoSmithKline	I	No data available
HPDC-T cells	HB-Vac	Sun Yat-Sen University	I/II	No data available
AIC649	Inactivated parapox virus	AiCuris	I	(134)
GS-9620	TLR-7 agonists	Gilead Sciences	II	(135, 136)
RO7020531,RG7795(ANA773), RG7854	TLR-7 agonists	Roche	I	No Results Posted
JNJ-64794964	TLR-7 agonists	Janssen	I	(137)
GS-9688	TLR-8 agonists	Gilead Sciences	II	No data available
SB 9200	RIG-I agonists	Spring Bank Pharmaceuticals	I	No data available
GS-9992	RIG-I agonists	Gilead Sciences	II	No data available
γδT Cells	–	Jinan University Guangzhou	I	No data available
Nivolumab	Anti-PD-1	Gilead Sciences	Ib	(138)

### GS-4774

GS-4774 is a recombinant yeast-based vaccine that contains HBV-specific antigens such as HBx protein and large HBsAg (126). Its safety, tolerability, and immunogenicity have been verified in normal healthy individuals. However, GS-4774 showed no clinical benefit in virally suppressed individuals with CHB in a Phase II study. There was no significant decrease in mean HBsAg levels, with ≥0.5 log10 IU/ml reductions in HBsAg in only three patients administered a high GS-4774 dose of 40 YU, and no case of HBsAg clearance. Five HBeAg-positive individuals administered GS-4774 had HBeAg loss, with none recorded among control patients (127).

Recently, the results of an open-label, multicenter, randomized study (http://clinicaltrials.gov no: NCT02174276) of CHB patients using tenofovir disoproxil fumarate (TDF) alone or combined with GS-4774 were published (128). Significantly increased IFN-γ, TNF, and IL2 production was evidenced in HBV-specific CD8^+^ T cells from individuals administered GS-4774 and TDF at weeks 24 and 48, but not in those under TDF monotherapy. Increased T-cell functions were correlated with reduced numbers of regulatory T cells. Again, GS-4774 treatment resulted in no reduction of HBsAg levels in patients. Thus, GS-4774 is able to stimulate host CD8^+^ T cell responses but not sufficient to control HBV.

### NASVAC

The nasal vaccine candidate (NASVAC) is composed of HBsAg and HBcAg. In Phase I experiments, nasal spray with NASVAC was shown to induce anti-HBcAg antibodies in all subjects 30 days after administration of 3 doses. Seventy-five percent of the tested subjects developed anti-HBsAg antibodies at the latest time point of 90 days upon the start of vaccination (139). NASVAC was also well-tolerated after intramuscular application, with all the 14 enrolled patients developing HBV-specific lymphoproliferative responses. A total of 80 CHB patients were administered NASVAC intranasally or subcutaneously in a Phase III, randomized, controlled clinical trial. Compared with Peg-IFN treatment, NASVAC therapy resulted in a similar proportion of patients with viral load under the detection limit at the end of treatment (59.0 vs. 62.5%, *p* > 0.05). A higher percentage (57.7% vs. 35.0%) of patients had sustained HBV load under the detection limit at 24 weeks of follow-up (130). However, these results need to be verified in future clinical trials.

### YIC

The HBsAg-hepatitis B immunoglobulin (HBIG) complex (YIC) has been tested in HBeAg-positive CHB patients in a Phase II clinical trial with six doses. Compared with the control group administered alum only, the patients vaccinated with YIC showed improved HBeAg seroconversion (9% in the control group vs. 21.8% in the YIC group), enhanced anti-HB production, and decreased viral load (140, 141). However, a Phase III clinical trial with 12 doses failed to show satisfying results (142). Indeed, the trial exploring the immunological mechanisms of YIC (clinical registration number: ChiCTR-TRC-11003189) showed that CHB patients immunized with YIC in combination with adefovir treatment exhibit increased CD4^+^ and CD8^+^ T cell responses. A significant increase in IFN-γ production and reduced expression of inhibitory factors including IL-10, TGF-β, and Foxp3 were detected in CD4^+^ T cells from individuals immunized with YIC (143).

### HepTcell

HepTcell is a mixture of nine synthetic peptides comprising HBV-specific T-cell epitopes. In a Phase I trial performed in the United Kingdom and South Korea, HepTcell was tested in HBeAg-negative CHB cases treated with entecavir or TDF. Three monthly injections of HepTcell were well tolerated and induced cellular immune responses against HBV antigens in patients (131). A Phase II trial is anticipated to start in 2020 to evaluate the immune responses of HepTcell with more injections in an expanded patient cohort with chronic HBV infection.

### DNA Vaccines

At the moment, a number of clinical studies on DNA-based HBV vaccines are still ongoing (Table 1). In an early French Phase I/II clinic trials, therapeutic DNA vaccines have been confirmed as safe in CHB patients (132, 133). However, satisfying results have not been obtained even in combination with NUCs, with only transient/weak T cell responses, increase in NK cells, and no sustained virological responses. In HBV carriers receiving lamivudine treatment, a DNA vaccine containing the majority of HBV genes in addition to IL-12 DNA could induce HBV-specific IFN-γ secreting T-cells, maintained for 40 weeks or more upon therapy and correlating with virological responses (144). A non-replicative adenoviral vector harboring HBsAg, HBcAg, and Polymerase (TG1050) was shown to induce strong HBV multi-specific and prolonged T-cell responses in the mouse model (145). A Phase Ib study was performed in CHB patients and demonstrated the safety and immunogenicity of TG1050, supporting future testing in combination with antivirals (146).

### The Anti–PD-1 Antibody Nivolumab

In a Phase Ib study (ACTRN12615001133527), the anti–PD-1 antibody nivolumab was tested at a single dose in HBeAg-negative CHB cases with viral suppression, either alone or in combination with GS-4774. Reduced HBsAg levels were detected in all 22 individuals administered 0.3 mg/kg nivolumab alone or with GS-4774 at week 12. Interestingly, 2/10 patients in the GS-4774 + nivolumab group and 1/12 of the nivolumab monotherapy group had serum HBsAg reductions ≥ 0.5 log10 IU/ml at 24 weeks. A single individual with significantly decreased HBsAg levels in the nivolumab arm showed HBsAg loss at week 16, and anti-HBsAg responses at 10 weeks upon trial completion with anti-HB titers surpassing 500 IU/L 12 months after treatment (138).

### Oral TLR-7/8 Agonists

There are currently several oral TLR-7/8 agonists in clinical trials, including GS-9620, RO7020531, RG7795 (ANA773), RG7854, JNJ-4964 (AL-034/TQ-A3334), and GS-9688 (Table 1). GS-9620 represents an effective, selective, and orally active TLR7 agonist. Its safety has been confirmed in treatment-naïve or currently treated individuals with chronic HBV infection. No marked circulatory IFN-a increase and associated symptoms were observed, although ≥2-fold ISG15 increase was evidenced in serum samples from individuals administered 2- or 4-mg GS-9620 (136, 147). Twelve-week GS-9620 administration in CHB patients with HBV well suppressed by NUCs resulted in increased T- and NK-cell responses and decreased NK cell-mediated T cell inhibition, while no significant decrease in serum HBsAg levels was achieved. The beneficial effect of GS-9620 in strengthening HBV-specific immune responses needs to be validated with a longer assessment period or combination with IFN therapy (148). In another Phase II study, GS-9620 treatment of naïve CHB patients, even after combination with tenofovir (TDF), did not significantly decrease HBsAg levels (135). Another TLR 7 agonist, RO7020531, is being assessed in a Phase I study.

### AIC649

AIC649 is a patented inactivated parapox virus (iPPVO) and a novel biological immunomodulator. AIC649 could induce natural, self-limiting immune responses and boost immune responses to unrelated pathogens. A preclinical study in the woodchuck model demonstrated that AIC649 administration leads to a significant decrease in WHsAg even after cessation of treatment (149). Continuous WHsAg suppression as well as anti-WHsAg antibodies and cell-mediated immune responses were measured in combination with ETV (Poster AASLD 2017-10-24), indicating a potential for treatment of chronic HBV infection.

### DC/DC-CIK

Clinical examination indicated that HBsAg-pulsed DCs induce anti-HBsAg antibody and HBsAg-specific cellular immunity in 2/5 and 1/5 CHB patients, respectively, without obvious liver damage. Anti-HB antibodies were detectable 1 month after administration of HBsAg-pulsed DCs and increased progressively for 5 months in one patient (105). HBsAg-pulsed DC vaccines could accelerate HBsAg clearance as well as HBsAg/HBsAb seroconversion in patients with low HBsAg levels (Meng et al. unpublished data). Autologous DC-vaccines could efficiently inhibit HBV replication, decrease the viral load, clear HBeAg, and induce HBeAg/anti-HBe seroconversion, even leading to loss of HBsAg (150). However, randomized controlled studies are required for further validation of DC vaccines for treatment of CHB.

In a clinical study, over 2 log 10 fold decrease in HBV load was detected in 21/33 (63.6%) of CHB patients administered HBsAg activated autologous DC-CIK (151). CIK without HBsAg pulse could also reduce serum HBV load and promote HBeAg clearance in patients with high ALT levels. At 36 weeks of follow-up, HBeAg negativity and HBeAg seroconversion were found in 33.3 and 9.5% of CHB patients who received CIKs, respectively (152).

Yet, clinical trials based on immunotherapy did not show satisfactory results. The trials with DNA vaccines and YIC failed to deliver positive results, reducing the hope placed in immunotherapy. However, the rather negative results in these studies are not surprising as therapeutic immunomodulation is not simply the induction of adaptive immunity but needs to tackle negative immune regulation established during chronic viral infections. It is important to test these approaches in cohorts of patients under antiviral treatment and suppressed HBV replication. In such patients, the host immune system may recover to some extent from HBV-mediated impairment and respond more robustly to immune stimulation. There are patients with sustained low HBV loads, which may hint to naturally enhanced immune control of HBV infection. It would be useful to select such patients for future clinical trials. There are still a great number of options and combinations of these approaches to be considered, along with new innovations from future research.

## IFN-α-Based Immunotherapy Plays an Important Role in HBV “Cure” in Individuals With Functional Intrinsic Immune Responses

PEG-IFN-α possesses antiviral and immunomodulatory effects, and remains the most effective drug for the treatment of CHB patients. In the treatment of naïve CHB patients, Peg-IFN-α administration for 48 weeks achieves superior efficacy over lamivudine, as reflected by HBeAg seroconversion, HBV DNA clearance, and HBsAg seroconversion. Although only 4% of HBsAg loss was reported at 6 months off-therapy, this rate reached 11% after 4 years of follow-up (153, 154).

PEG-IFN-α has been demonstrated to enhance HBsAg loss, especially in patients administered NUCs with HBsAg titers of <1,000 IU/ml. HBsAg clearance rates at 48 weeks were 9% (2 out of 22) and 15% (4 out of 26), in switch-to and add-on therapy CHB patients, respectively (155). A systematic review revealed that CHB patients treated with NUCs for at least 48 weeks are more likely to achieve HBsAg loss (11%), using a PEG-IFN-α-based combination treatment (10). HBsAg loss occurred significantly often in selected CHB patients in a new SWITCH study with initial administration of NUCs and switch to PEG-IFN-α-2a (155). Another study demonstrated the effectiveness of PEG-IFN-α-2a for the treatment of inactive HBsAg carriers, resulting in high rates of HBsAg depletion and seroconversion (9). PEG-IFN-α treatment showed enhanced HBsAg seroconversion rate in CHB cases with low HBsAg and HBV DNA levels. Therefore, more clinical trials (e.g., NCT02745704, NCT02893124, and NCT02838810) are currently on the way to identify the optimal usage of Peg-IFN-α treatment in CHB patients with low HBsAg levels. Peg-IFN-α may contribute significantly to the cure of HBV infection if diversely integrated in multi-drug regimens, e.g., with lower dosage or intermittent application to avoid severe adverse effects.

Peg-IFN-lambda (Peg-IFN-λ), a type-III IFN, has been attributed dual immunomodulatory effects on both innate and adaptive immune responses in chronic HBV infection. IFN-λ shares similar ISG induction pathways as IFN-α, and Peg-IFN-λ exerts antiviral effects similar to those of Peg-IFN-α. Interestingly, Peg-IFN-λ showed substantially improved tolerability than Peg-IFN-α, since IFN-λ binds to type III interferon receptors, which are restricted to cells of epithelial origin, including hepatocytes (156, 157). Thus, the clinical application of Peg-IFN-λ may benefit CHB patients.

## Conclusions and Future Perspectives

Chronic HBV infection is considered a result of HBV-specific immune tolerance. Based on this concept, breaking immune tolerance and restoring HBV-specific immune responses may ultimately lead to HBV control and clearance in patients. It becomes possible to induce HBV-specific immune responses in patients, yet not with the desired results of HBV control. Immunotherapeutic approaches are also hampered by the risk of overshooting immune responses in CHB patients and causing uncontrolled liver damage. Thus, combinations of potent antiviral treatment and carefully adjusted immune modulation may achieve a “cure” of CHB, without severe liver damage and disease progression. Nevertheless, HBV-specific CAR-T/TCR-T cells in combination with checkpoint inhibitors may be a potential strategy for HBV control.

In the past years, an essential role for HBV-specific T cell responses in viral control has been emphasized. Though functional T cell response is required for HBV control, it is definitely not sufficient for a successful immunotherapeutic approach. Given that the various therapeutic vaccines tested so far were highly effective in priming specific T and B cell responses to HBV antigens, they generally do not achieve significant and long-lasting viral suppression in animal models and patients. These rather disappointing results may have diverse reasons but the potential conceptual problem should not be ignored. Transfer of large numbers of activated CD8^+^ T cells to HBsAg in HBV Tg mice only led to transient suppression of HBV replication (158). Transfer of splenocytes from HBsAg-vaccinated mice to HBsAg Tg mice resulted in sustained production of anti-HBsAg antibodies and HBsAg clearance in the peripheral blood of recipient mice (159). However, HBsAg-specific CD8^+^ T cells became undetectable, while HBsAg production in the liver continued in recipients. Importantly, no inflammation and T cell infiltration in the liver of Tg mice were observed. Further, HBV-specific T cells could be detected in mice with persistent HBV replication after hydrodynamic injection, but did not enter the liver unless an intrahepatic immune activation was triggered by TLR3 stimulation (160). A recent report also showed that HBV-specific T cells are detectable in the peripheral blood of young patients in the immune tolerant phase (103, 161). HBV-specific T cells were found to possess the ability to proliferate and produce cytokines (162). Specific CD8^+^ T cells in all mentioned cases apparently could not cause chronic liver inflammation, consistent with the findings reported by other immune transfer studies (163). Therefore, the antiviral effects of HBV-specific T cells may require appropriate conditions in the liver. TLR agonists may be useful for the promotion of T cell functions in the liver (160, 164, 165), by recruiting various immune cells into the liver to form tertiary lymphoid structures (166–168). These aspects have been discussed in recent reviews and need to be investigated in future studies (32, 52, 58, 92).

Beside T cell-mediated antiviral effects, other mechanisms for HBV control need to be considered in future approaches. The roles of other immune cell types are not yet well studied in the context of HBV immunity. NK cells may contribute significantly to HBV control during acute and chronic infection (22, 169, 170), playing an important role for successful IFN-α therapy (171, 172). At the moment, modulation of the NK cell activity to control HBV infection has only been tested in few studies and needs more attention (173). Recently, the function of HBV-specific B cells in HBV infection has been characterized by using fluorescently labeled HBV proteins, showing impairment in chronically infected patients (174–176). Modulation of B cell function and antibody production may represent another option for immunotherapy (87, 177). A number of host genetic determinants have been identified to contribute to HBV control and pathogenesis (178). Many of these determinants play a role in immune control of HBV infection. However, there are other factors such as UBE2L3 gene that regulate HBV replication by controlling cccDNA stability and yet unknown processes (179). By comparing hepatic gene expression profiles in patients from different phases of the natural course of chronic HBV infection, a number of differently expressed host genes were found to contribute to HBV control in inactive carriers with low HBV loads (180). This result is somewhat relevant as no immune-related gene was actually found to be active in such patients, indicating that immune mechanisms may not be effective in patients with low HBV replication and gene expression. This is rational as antigen-specific T cells may target infected cells only with sufficient levels of antigen production and would ignore hepatocytes with HBV production below the recognition threshold. Apparently, there are diverse non-immune host restrictions for HBV replication that are important for achieving long-term, non-cytotoxic HBV control.

While we appreciate the great relevance of host immune responses for HBV control, successful therapy of chronic HBV infection may require combinations of antiviral treatment with NUCs, activation of intrahepatic innate immunity, stimulation of specific T cell responses, and finally switching on of non-immune mechanisms for sustained HBV control without undesired side effects. Recent findings pointed out the central role of cccDNA and/or integrated HBV DNA in hepatocytes for “functional cure” of chronic HBV infection. It is yet unknown whether HBsAg seroconversion/clearance alone would protect against HCC with cccDNA or integrated HBV DNA still present in hepatocytes. Likely, immunotherapeutic approaches may clear at least a great part of infected hepatocytes with cccDNA and/or integrated HBV DNA if they express HBV proteins, thereby reducing the risk of HCC development. These aspects deserve further investigation.

## Author Contributions

ZM searched the literature, performed review design, and wrote the manuscript. YC designed the figures and contributed to manuscript preparation. ML contributed to review conception and manuscript revision.

### Conflict of Interest

The authors declare that the research was conducted in the absence of any commercial or financial relationships that could be construed as a potential conflict of interest.
